# Serotyping and Cross-Reactivity's Between Different *Pseudomonas aeruginosa* Isolates Prevalent in Iran

**Published:** 2010-06

**Authors:** H Ahmadi, S Maleknia, B Tabaraie, D Norouzian, F Poormirza-gholi, M Nejati, MH Hedayati, MR Beik Mohammadi, A Behnoodi, M Izadpanahi

**Affiliations:** 1 Department of Bacterial Vaccines and Antigen Production, Pasteur Institute of Iran, Tehran, Iran; 2 Department of Microbiology, Islamic Azad University, Zanjan Branch, Iran

**Keywords:** *Pseudomonas aeruginosa*, agglutination test, cross-reactivity, antigenic schema

## Abstract

**Background and Objectives:**

300 *Pseudomonas aeruginosa* strains were isolated from hospitalized patients in Iran. Using international antigenic typing system (IATS) antibodies, all strains were classified into 16 serotypes while serotype 14 was not identified among the 17 known serotypes. To evaluate the rate of cross-reactivity between O- antigenic determinants, monospecific polyclonal antibodies were made against whole-killed-cells and live cells of each serotype.

**Materials and Methods:**

Each antiserum was challenged against homologous and heterologous antigens using slide agglutination test. The degree of agglutination reaction is shown by –ve, 1+ve, 2+ve, 3+ve and 4+ve for 0, 25%, 50%, 75% and 100% agglutination respectively. Then, the results were tabulated for further study.

**Results:**

The rate of cross-reactivity between O-antigenic determinants demonstrated that strains 10.55 and 15.14 had the highest agglutination reaction with serum of all the homologous and heterologous serotypes.

**Conclusion:**

Evaluation of the results obtained from the present study can be applied in production of reliable vaccines and antisera as therapeutic agents or as diagnostic kits.

## INTRODUCTION

*Pseudomonas aeruginosa* is one of the most common opportunistic pathogen of nosocomial infections (1–9). Being amphibiotic in nature (parasite & saprophyte), *P. aeruginosa* causes a high epidemic spread in wound and burn infections and in patients with immune deficiency syndrome, neoplasia, cystic fibrosis, ones who have undergone surgery, organ transplantation or have received artificial organs (2, 3, 5, 6, 9, 10).

Based on the antigenic specification of the oligosaccharide side chain of LPS (O-Antigen), many serological classification systems for *P. aeruginosa* were proposed (11–14), but the most reliable typing system was the one suggested by the International Committee of Microbiology (ICM) in 1970 which adapted 17 heat stable O-antigenic typing system abbreviated by arabic numbers 1 through 17 (1, 7).

In the present study, we have performed the slide agglutination test according to the panel of the ICM typing system, using 300 pathogenic *P. aeruginosa* isolates collected from different hospitals in Iran to suggest a new model of antigenic schema for *P. aeruginosa* serotyping so that, to control or prevent the infection.

## MATERIALS AND METHODS

**Bacterial strains**. Applying biochemical tests and using standard somatic typing antisera from Difco (Franklin Lakes, NJ USA) & Denka Seiken (Tokyo, Japan) Companies, all 300 clinical isolates of *P. aeruginosa* were classified among the Iranian isolates. Each serotype was lyophilized, encoded and kept as stock culture in the Collection of Standard Bacteria of the Pasteur Institute of Iran (CSBPI) till use. Standard strain of *P. aeruginosa* PTCC-1074 was used as a positive control.

**Antisera preparation.** Each *P. aeruginosa* serotype was grown on Heart Infusion Agar (HIA) (MERK, Darmstadt, Germany) for 18 hours at 37°C. Cells were harvested by PBS (Phosphate Buffer Saline, pH = 7.2) containing 0.5% phenol and 2% (V/V) of 20% glucose solution. Each suspension was heated at 90°C for one hour in shaking water bath and then washed three times with the same buffer. A portion of each cell suspension was adjusted to 9×10^8^ cells/ml in sterile PBS (pH = 7.2) and then used as immunizing antigen.

A group of 2 white New Zealand rabbits, weighing 1.5 to 2 Kg, were immunized intravenously with each serotype suspension in increasing doses of 0.25, 0.5, 1, 1.5 and 2 ml at 4 day intervals 7 days after the last injection. The sera were collected from each group and pooled. After addition of 1:10000 (W/V) thiomersal, all sera were kept at 4°C till use.

**Rapid slide agglutination test.** This test was applied for both the live and heat killed cells of all 16 isolates. PBS also used as negative control. Two drops of each antiserum was placed on a clean glass slide. A loop full of 18 hours growth from each live serotype was mixed evenly with the first drop and one drop of a thick suspension of each heat-killed cells was mixed second drop, slides were tilted by hand and the rate of agglutination reaction was recorded from four positive (4+ means strong agglutination appearing in a few seconds and one positive means a week agglutination reaction at the end of one minute) (19).

The results were tabulated so that the rate of agglutination of both live and killed bacteria from homologous and heterologous strains against each serum was recorded.

## RESULTS

Comparison of the results observed in Table 1 & 2 show a minor antigenic difference between the live and killed antigens. It was also observed that strains 10.55 and 15.14 had the highest agglutination reaction with serum of all the homologous and heterologous serotypes except serotype 8 and 9. Strain 1.101 had weak agglutination reaction. The sera against strains 6.95, 6.109, 7.107, 17.110 had maximum agglutination reaction with homologous and heterologous strains. Strains 11.106 and 15.14 showed the minimum agglutination reaction.

**Table 1:** Antigenic schema with live organism antigens invitro by the slide agglutination test.

Antiserum	1:101	2:160	3:172	4:89	5:60	5:111	6:95	6:109	7:107	7:197	8:98	9:105	10:55	11:106	12:80	12:159	13:108	15:14	16:190	17:110	PTCC–1074
Antigen																					
1:101	4+	2+	2+	–	–	–	–	–	–	2+	2+	–	2+	–	–	–	–	–	–	2+	4+
2:160	–	4+	1+	–	4+	–	1+	–	–	2+	–	–	1+	–	–	–	4+	–	4+	–	–
3:172	–	–	4+	–	–	4+	–	–	–	4+	–	–	3+	–	–	–	–	–	–	1+	–
4:89	–	–	–	4+	–	–	3+	1+	4+	–	4+	4+	–	–	–	–	–	–	–	–	–
5:60	1+	3+	–	1+	4+	1+	–	–	–	–	–	–	3+	–	3+	3+	–	–	–		–
5:111	–	4+	–	–	4+	4+	–	–	–	–	–	–	–	–	–	–	4+	–	4+	–	–
6:95	–	–	–	–	–	–	4+	4+	4+	–	4+	4+	–	–	–	–	–	–	–	4+	–
6:109	–	–	–	4+	–	–	4+	4+	4+	–	4+	+4	–	–	–	–	–	–	–	4+	–
7:107	–	–	–	3+	–	–	3+	4+	4+	–	4+	4+	–	–	–	–	–	–	–	4+	–
7:197	–	–	4+	–	–	–	2+	–	–	4+	–	–	–	–	–	–	–	–	–	–	–
8:98	1+	–	–	4+	–	–	4+	4+	4+	–	4+	4+	–	–	–	–	–	–	–	4+	–
9:105	3+	–	3+	–	1+	3+	–	1+	–	–	1+	4+	1+	–	–	–	–	–	–	2+	
10:55	4+	4+	4+	4+	4+	4+	–	4+	4+	4+	1+	4+	4+	–	–	–	–	2+	–	4+	4+
11:106	–	–	–	–	–	–	–	–	–	–	–	–	4+	4+	–	–	–	–	–	–	–
12:80	–	–	–	–	–	–	–	–	–	–	–	–	–	–	4+	4+	–	–	1+	–	–
12:159	–	–	–	–	–	–	–	–	–	–	1+	2+	–	–	4+	4+	–	2+	1+	–	–
13:108	–	–	–	–	4+	4+	–	–	–	–	–	–	–	–	–	–	4+	–	4+	–	–
15:14	3+	4+	2+	4+	4+	4+	4+	4+	4+	1+	–	–	4+	4+	4+	4+	4+	4+	4+	4+	3+
16:190	1+	–	–	–	3+	1+	4+	4+	3+	1+	–	1+	–	4+	–	–	4+	4+	4+	–	3+
17:110	1+	–	–	4+	–	–	4+	4+	4+	–	4+	4+	–	–	–	–	–	4+	–	+4	–
PTCC–1074	4+	4+	4+	–	4+	4+	–	–	4+	2+	4+	3+	–	–	–	–	–	3+	–	–	+4

## DISCUSSION

Numerous epidemiological studies have revealed that more than 10% of all nosocomial infections and 11% of all organisms isolated from blood culture were *P. aeruginosa.* This rate increases to 30% in cancer and burn-wards and mortality rate among patients with septicemic infection rises to 80% (11). Resistance to different types of antibiotics and failure in response to drug treatment against *P. aeruginosa* infection has led to applying different ways of treating against this microorganism (17). Immunotherapy by vaccination or anti- *P. aeruginosa* hyperimmune sera can be a reliable method in controlling the infection (17, 18).

Since the genetic structure of most *P. aeruginosa* serotypes are almost similar, determination of the extent of antigenic cross-reactivity between different serotypes leads us to design a reliable vaccine against the disease. Kusama et al (1978) in New York (1), Faure *et al* (2003) from California (5), Shigeta *et al* (1978) from Japan (7) and Liu (1987) from China (19) have reported classification and antigenic serotyping of *P. aeruginosa*. No vaccine production against *P. aeruginosa* has been reported so far in Iran and since the common strains of this organism are different from each other in different geographical locations in terms of antigenicity, pathogenicity and medical resistance, producing a vaccine from common strains in Iran can be taken into consideration. As it can be observed from Table 1 and 2, the three serotypes of 10.55, 15.14 and 8.98 or even just the first two serotypes of *P. aeruginosa* can be used in vaccine production. Due to the fact that these serotypes have shown the greatest antigenic similarities among the Iranian isolates of *P. aeruginosa*, moreover, they possibly can also be used in absorption of unwanted antibodies from hyper-immune sera against each serotype to produce mono-specific antisera which are used in serotyping of *P. aeruginosa* isolates.

**Table 2:** Antigenic schema with heat-stable somatic antigens invitro by the slide agglutination test.

Antiserum	1:101	2:160	3:172	4:89	5:60	5:111	6:95	6:109	7:107	7:197	8:98	9:105	10:55	11:106	12:80	12:159	13:108	15:14	16:190	17:110	PTCC-1074
Antigen																					
1:101	4+	2+	2+	–	1+	–	1+	–	–	2+	–	–	2+	–	–	–	–	–	–	2+	4+
2:160	-	4+	1+	–	4+	–	2+	–	–	2+	–	–	1+	–	–	–	4+	–	4+	–	–
3:172	2+	–	4+	–	2+	3+	2+	–	–	4+	–	–	2+	–	–	–	–	–	–	1+	–
4:89	–	–	–	4+	–	–	–	–	–	–	–	2+	–	–	–	–	–	–	–	–	–
5:60	4+	3+	–	1+	4+	1+	3+	1+	1+	–	1+	2+	2+	1+	–	–	1+	2+	–	1+	3+
5:111	–	4+	–	–	4+	4+	–	–	–	–	–	–	–	–	–	–	4+	–	4+	–	–
6:95	–	–	–	–	–	–	4+	4+	4+	–	4+	4+	–	–	–	–	–	–	–	4+	–
6:109	–	–	–	–	–	–	4+	4+	4+	–	4+	4+	–	–	–	–	–	–	–	4+	–
7:107	–	–	–	–	–	–	4+	4+	4+	–	4+	4+	–	–	–	–	–	–	–	4+	–
7:197	–	–	4+	–	–	–	–	–	–	4+	–	–	–	–	–	–	–	–	–	–	–
8:98	–	–	–	–	–	–	4+	4+	4+	–	4+	4+	–	–	–	–	–	–	–	4+	–
9:105	–	–	1+	–	–	2+	2+	–	3+	–	–	4+	1+	–	–	–	–	–	–	2+	–
10:55	4+	4+	4+	4+	3+	4+	4+	4+	4+	4+	3+	3+	4+	3+	–	–	3+	3+	–	4+	3+
11:106	–	–	–	–	–	–	–	–	–	–	–	–	4+	4+	–	–	–	–	–	–	–
12:80	–	–	–	–	–	–	–	–	–	–	–	–	–	–	4+	4+	–	–	1+	–	–
12:159	–	–	–	–	–	–	–	–	–	–	–	–	–	–	4+	4+	–	–	1+	–	–
13:108	–	4+	–	–	4+	4+	–	–	–	–	4+	–	–	–	–	–	4+	1+	4+	–	–
15:14	4+	4+	2+	4+	3+	4+	4+	4+	4+	1+	4+	3+	4+	4+	4+	4+	4+	4+	4+	4+	3+
16:190	–	4+	1+	–	4+	1+	–	–	–	1+	4+	–	–	–	–	–	4+	–	4+	–	–
17:110	–	–	–	–	–	–	4+	4+	4+	–	4+	3+	–	–	–	–	–	–	–	4+	
PTCC–1074	4+	–	–	–	–	–	–	–	–	2+	2+	–	–	–	–	–	–	–	–	–	+4
